# Transformer-Based Language Models for Group Randomized Trial Classification in Biomedical Literature: Model Development and Validation

**DOI:** 10.2196/63267

**Published:** 2025-05-09

**Authors:** Elaheh Aghaarabi, David Murray

**Affiliations:** 1 Office of Disease Prevention, National Institutes of Health, 6705 Rockledge Dr, Bethesda, MD, 20892, United States, 1 3014964000

**Keywords:** document classification, machine learning, natural language processing, randomized trials, transformer, AI, artificial intelligence, clinical trials, language model, development, dataset, biomedical, model, tool, trial, public health

## Abstract

**Background:**

For the public health community, monitoring recently published articles is crucial for staying informed about the latest research developments. However, identifying publications about studies with specific research designs from the extensive body of public health publications is a challenge with the currently available methods.

**Objective:**

Our objective is to develop a fine-tuned pretrained language model that can accurately identify publications from clinical trials that use a group- or cluster-randomized trial (GRT), individually randomized group-treatment trial (IRGT), or stepped wedge group- or cluster-randomized trial (SWGRT) design within the biomedical literature.

**Methods:**

We fine-tuned the BioMedBERT language model using a dataset of biomedical literature from the Office of Disease Prevention at the National Institute of Health. The model was trained to classify publications into three categories of clinical trials that use nested designs. The model performance was evaluated on unseen data and demonstrated high sensitivity and specificity for each class.

**Results:**

When our proposed model was tested for generalizability with unseen data, it delivered high sensitivity and specificity for each class as follows: negatives (0.95 and 0.93), GRTs (0.94 and 0.90), IRGTs (0.81 and 0.97), and SWGRTs (0.96 and 0.99), respectively.

**Conclusions:**

Our work demonstrates the potential of fine-tuned, domain-specific language models to accurately identify publications reporting on complex and specialized study designs, addressing a critical need in the public health research community. This model offers a valuable tool for the public health community to directly identify publications from clinical trials that use one of the three classes of nested designs.

## Introduction

Researchers need to identify publications from trials that use nested designs to access evidence relevant to community-level interventions and make informed decisions about public health strategies, as well as to understand the effectiveness of these interventions in improving health outcomes and reducing health disparities within populations. Additionally, they may require these publications to conduct meta-analyses and systematic reviews.

There are three important classes of nested designs widely used in clinical trials [1,2]. The parallel group- or cluster-randomized trial (GRT) involves the randomization of groups or clusters to study arms with observations taken using members of those groups or clusters [3-8]. This design is widely used to evaluate interventions that are delivered to groups or clusters that modify the physical or social environment, or that cannot be delivered to individuals without the substantial risk of contamination. The stepped wedge group or cluster-randomized trial (SWGRT) involves the randomization of groups or clusters to sequences; all groups or clusters begin in the control arm and transition to the intervention arm on a schedule determined by their sequence so that by the end of the trial, all groups or clusters are in the intervention arm [9]. The individually randomized group-treatment (IRGT) trial involves the random assignment of individuals to study arms but delivery of the intervention in a group-based format or using shared intervention agents [10,11]. All three nested designs have design, analytic, and sample size challenges not found in the traditional randomized clinical trial [1,2].

Currently, most public health researchers, including ourselves, use manual searches to identify GRTs, IRGTs, or SWGRTs, because, so far as we are aware, there are no automated methods for identifying published papers using these designs. However, manual searches can easily miss many qualifying publications due to the complexity of search parameters and the lack of consistent reporting. Document classification using machine learning and natural language processing techniques offers a more promising approach for categorizing documents into predefined groups.

Garcia et al [12] enhanced automatic document classification in the biomedical domain by leveraging Wikipedia knowledge to create bag-of-concepts representations, resulting in performance gains over traditional bag-of-words approaches in both single-label and multi-label classification tasks. Cohen [13] also proposed a biomedical text classifier, which integrates document words, MeSH terms, and normalized biological entity identifiers.

Previous studies have demonstrated the utility of machine learning approaches in identifying randomized controlled trials (RCTs) from biomedical literature databases. Marshal et al [14] used machine learning models, including convolutional neural networks, support vector machines, and ensemble models to identify RCT publications. Al-Jaishi et al [15] addressed the challenge of accurately identifying GRTs reports from bibliographic citations by leveraging static embedding techniques, developing and validating machine learning algorithms for information retrieval.

While these studies have focused on identifying specific types of RCTs, such as conventional RCTs and GRTs, our research aims to extend this approach to the identification of diverse categories of randomized trials, including GRTs, IRGTs, and SWGRTs. To achieve this, we propose a novel approach leveraging fine-tuned language models, specifically the pretrained BioMedBERT model, trained on a dataset of biomedical literature curated by the Office of Disease Prevention at the National Institute of Health.

Large language models represent an ideal choice for the development of biomedical text classifiers due to their capacity to grasp the contextual nuances within the data. Pretrained transformer language models, like bidirectional encoder representations from transformers (BERT) [16-18], have outperformed the existing deep neural network models, including convolutional neural networks and recurrent neural networks. Examples of transformer-based models trained on biomedical data include BioBERT [19], BioLinkBERT [20], BlueBERT [21], and BioMedBERT [22], which are pretrained on biomedical literature and clinical text.

While these pretrained models are readily applicable to common tasks due to their training on biomedical data, classifying scientific literature presents a unique challenge because scientific literature encompasses a diverse range of topics, writing styles, and research fields. Identifying and categorizing clinical trials into highly specialized categories is especially challenging, even for human coders. Leveraging transfer learning and fine-tuning a pretrained language model allows the machine learning platform to learn and adapt to the particular context and vocabulary of these types of documents, enhancing its effectiveness in tasks such as document classification, information extraction, and summarization [18].

To our knowledge, there is currently no transformer-based language model fine-tuned to identify clinical trial publications based on nested designs. In our method, we leverage BioMedBERT [22], a model that has been pretrained from scratch using abstracts from PubMed and full-text articles from PubMedCentral. We fine-tuned BioMedBERT using labeled clinical trials, with a specific focus on distinguishing various types of clinical trial publications, including GRTs, IRGTs, and SWGRTs. The fine-tuning process involved training on a carefully curated dataset comprising a substantial number of GRTs, IRGTs, and SWGRTs. The outcome of this fine-tuning process is a model that provides a high level of sensitivity and specificity in classifying and differentiating various types of randomized trial publications.

## Methods

### Traditional Machine Learning Models (Baseline Model)

In our study, we initially established a baseline model for classifying publications using traditional machine learning and word embedding techniques to demonstrate the effectiveness of employing a transformer-based model in identifying publications based on nested designs. To create the baseline model, we employed FastText (Facebook AI Research) to generate word embeddings, followed by a logistic regression model. Logistic regression has been widely recognized in the literature as an effective classifier for text data due to its simplicity, interpretability, and robust performance across various domains [23]. To enhance the model’s capability to process biomedical text data, we leveraged pretrained FastText embeddings specifically trained on PubMed and MIMIC-III data, known as BioWordVec [24], to extract meaningful features from titles and abstracts of publications. Subsequently, we used these extracted embeddings to train a logistic regression model for the classification of publications.

### Evaluation Metrics

There are various metrics available to evaluate the performance of a classifier. While the area under the curve is commonly used in binary classifiers, accuracy can be helpful for balanced evaluation datasets. However, in our case, where the model serves as a multiclass classifier with imbalanced data, the F_1_-score emerges as the most reliable metric [25]. Our primary objective was to fine-tune an existing pretrained language model to maximize the F_1_-score on a validation dataset.

The F_1_-score is the harmonic mean of precision and recall. Precision is the ratio of correctly predicted positive observations to the total predicted positives. It measures the accuracy of the positive predictions made by the model. High precision ensures that when our model predicts an article belongs to a nested design group, it is highly likely to be accurate. This is crucial in applications where precision contributes to the trustworthiness of the classification outcomes, such as systemically classifying research publications. Recall, which is the same as sensitivity, is vital when the cost of false negatives is high. A high recall ensures that our model effectively captures a comprehensive set of articles within each predefined group.

The F_1_-score is particularly useful in situations where there is an imbalance between the classes or when there is an equal importance placed on precision and recall. It is a metric that balances the trade-off between precision and recall, providing a single value that reflects the overall performance of the model.

Our dataset is imbalanced with fewer examples in the IRGT and SWGRT classes, and accuracy alone can be misleading. Precision, recall, and F1-score provide a more nuanced view of a model’s effectiveness, especially in identifying the strengths and weaknesses associated with false positives and false negatives.


$$\text{Precision}=\frac{\text{True Positives}}{\text{True Positives}+\text{False Positives}}$$

$$\text{Recall}=\frac{\text{True Positives}}{\text{True Positives}+\text{False Negatives}}$$

$$\text{F1 Score}=2\times \frac{\text{Precision}\times \text{Recall}}{\text{Precision}+\text{Recall}}$$

Specificity serves as a crucial metric for assessing the number of false positives within a model. It measures the ability of the model to correctly identify negative instances, thus providing insight into the model’s performance in avoiding false-positive predictions. Although it was not initially used as an evaluation metric during our experimental phase and model development, it was calculated to provide a comprehensive assessment of performance, particularly in gauging the rate of false positives.


$$\text{Specificity}=\frac{\text{True Negatives}}{\text{True Negatives}+\text{False Positives}}$$

The weighted average considers the class imbalance in the dataset by considering the contribution of each class proportional to the number of instances in that class. In other words, classes with more instances have a greater impact on the overall metric than classes with fewer instances.


$$WeightedAverage=\frac{\sum_{i=1}^{C}m_{i}∙n_{i}}{N}$$

Where

$m_{i}$ is the metric value (eg, precision, recall, F1-score) for class, $n_{i}$ is the number of instances in class i, C is the number of classes, and *N* is the total number of instances in the dataset

### BioMedBERT

We chose BioMedBERT as the initial pretrained model due to its superior performance compared to other existing models on our gold standard data, publications published prior to 2021 that were identified as GRT, IRGT, and SWGRT papers using search queries. BioMedBERT is a pretrained language model developed by Microsoft Research for biomedical text processing using abstracts from PubMed and full-text articles from PubMedCentral [22]. It is a specialized variant of the BERT architecture [16], designed to capture domain-specific nuances in biomedical literature. The architecture of BioMedBERT enables it to learn contextualized representations of words and phrases bidirectionally and understand the contextual relationships within biomedical texts. The BERT model used in our study was downloaded from Hugging Face’s Transformers library, configured as a classifier. In this framework, the tokenizer automatically manages special tokens such as [CLS] and [SEP], ensuring proper preprocessing for input sequences.

For the classification task, the [CLS] token's embedding from the final layer of BERT serves as a representation of the input sequence's contextual information. A multilayer perceptron is applied to this embedding to perform the classification. This multilayer perceptron consists of fully connected layers that map the [CLS] token's representation to the output space, followed by the softmax activation for probability distribution over classes.

The pretrained BioMedBERT uses a bidirectional transformer architecture with several layers of self-attention mechanisms. The model embeddings, including word embeddings and positional embeddings, contribute to encoding patterns in biomedical language.

During the pretraining phase, BioMedBERT was initialized with weights obtained from training on a domain-specific corpus: 14 million abstracts, 3.2 billion words, and 21 gigabytes [22]. This large-scale training ensures that the model captures a wide range of biomedical concepts, terminology, and contextual relationships. BERT-BASE [16] with 12 transformer layers and 100 million parameters was used to pretrain BioMedBERT.

A pooling layer was introduced atop the transformer’s final layer, known as the embedding layer. This embedding layer underwent pooling to derive a fixed-size representation of the entire input sequence. To capture nonlinearity and intricate patterns within the data, a feedforward layer was incorporated. This layer is linked to the output layer, responsible for computing logits.

### Fine-Tuning BioMedBERT

For this study, we fine-tuned BioMedBERT to adapt the model to the specific nuances of our dataset related to publications from clinical trials that use nested designs. By leveraging the contextual information encoded in BioMedBERT, we aimed to enhance the accuracy and efficiency of our machine learning model in identifying and distinguishing various types of clinical trial publications. In particular, our goal was for BioMedBERT to serve as a multiclass classifier that can categorize biomedical publications into four distinct categories: GRT, IRGT, SWGRTs, and the broader category of publications based on studies that used other designs, which we refer to as negatives (Multimedia Appendix 1).

### Data

The National Institute of Health Office of Disease Prevention provided a labeled dataset consisting of publications from PubMed, published prior to 2021, with each publication categorized into one of the three classes: GRT, IRGT, and SWGRT. We selected nonclinical trial publications from a list of 120 journals that published most of the nested clinical trial publications; we will refer to those publications as negative publications. The original dataset consisted of 891 GRT publications, 59 IRGT publications, 109 SWGRT publications, and 996 negative publications. The first version of the fine-tuned language model underwent training on titles and abstracts from this dataset. The best-performing model was subsequently employed to classify unlabeled publications published in 2021. After thorough verification of predictions by domain experts, a complementary set of 299 GRT, 40 IRGT, 65 SWGRT, and 1200 negative examples from 2021 was added to the original training, validation, and test sets.

The new dataset served as the foundation for tuning the model hyperparameters to predict labels for publications published in 2022. With the improved training data, the model demonstrated higher accuracy and F1-score, enabling precise classification of publications from the subsequent year, 2022. The same strategy as above was employed to add 2022 verified data to our training dataset, which resulted in the addition of 461 GRT, 195 IRGT, and 93 SWGRT, and 539 negative publications to the training, validation, and test datasets to prepare the classifier to predict labels for publications published in 2023. The Results section describes the final performance of the model trained and evaluated on this dataset.

### Tokenization

To process the textual data, we utilized the Hugging Face Trainer API in conjunction with the BioMedBERT tokenizer. The BioMedBERT tokenizer is trained on a corpus of biomedical text to tokenize and segment text into subwords using the WordPiece algorithm, just like the original BERT tokenizer. Therefore, the tokenizer is tailored to handle biomedical terminology and language patterns [22]. For instance, when tokenizing the title “Comparison of different intervention methods to reduce the incidence of venous thromboembolism: study protocol for a cluster-randomized, crossover trial” from a 2023 publication, the tokens are segmented as follows: [“comparison,” “of,” “different,” “intervention,” “methods,” “to,” “reduce,” “the,” “incidence,” “of,’ “venous,” “thromboembolism,” “:,” “study,” “protocol,” “for,” “a,” “cluster,” “-,” “randomized,” “,”, “crossover,” “trial,” “.”]. The tokenization process was carried out separately for the titles and abstracts of the publications. In order to maintain computational efficiency and manage memory constraints, we imposed a length limit on the tokenized text. Titles were truncated to a maximum length of 30 tokens, which was the maximum title length in the data, while abstracts were truncated to 256 tokens from the start of the text, ensuring that majority of abstracts in our dataset fit within this allocation. Shorter sequences were padded with zeros. Based on the length distribution of titles and abstracts, we adjusted the allocation to align with actual usage patterns, ensuring that all instances remain within the supported range while optimizing model efficiency.

### Addressing Class Imbalance

An imbalanced dataset refers to any dataset where there is an unequal distribution among classes, with one or more classes having significantly fewer instances than others. When trained on imbalanced datasets, models may exhibit a bias towards predicting the majority class more frequently, resulting in poor generalization for the minority class. Therefore, selecting the appropriate evaluation metric becomes crucial in such scenarios. A model might achieve high accuracy by predominantly predicting the majority class while displaying poor performance on the minority class. Various strategies exist to address class imbalance, including selecting appropriate performance metrics, such as precision-recall or the F_1_-score, to accurately reflect model performance. Techniques like undersampling the majority class, oversampling the minority class, employing synthetic data generation methods like SMOTE (synthetic minority over-sampling technique), leveraging algorithms designed to handle class imbalance robustly, and incorporating cost-sensitive learning by assigning costs to the loss function are among the approaches commonly employed [26-28]. Given the observed class imbalance within the dataset, we implemented a customized loss function to mitigate the impact of this imbalance during the training phase. To achieve this, inverse class weights were calculated based on the number of examples in each class within the training dataset. These weights were then utilized in the weighted categorical cross-entropy loss function, assigning varying levels of importance to each class during model training.

The weighted categorical cross-entropy loss function is defined as follows:


$$\text{Weighted Categorical Cross-Entropy Loss}=-\sum_{i=1}^{N}\sum_{j=1}^{C}w_{j}\cdot y_{i,j}\cdot \log\left(P_{i,j}\right)$$

where C is the number of classes, N is the number of samples in the dataset, $y_{i}$ is the true probability distribution (one-hot encoded vector) for class i, $P_{i}$ is the predicted probability distribution for class i, and $w_{i}$ is the weight assigned to class i

The inverse class frequency method is used to assign higher weights to classes with fewer examples. The rationale is to give higher weights to classes that are under-represented, making the model more sensitive to minority classes and potentially improving performance on imbalanced datasets.


$$w_{i}=\frac{\text{Total Number of Examples}}{\text{Number of Examples in class }i}$$

### Hyperparameter Tuning

To evaluate the model’s performance, we split our dataset into three subsets: a training set, a validation set, and a test set. The test set comprised 20% of the total dataset and was created by stratified random sampling from the labeled data. To assess the generalizability of our model to unseen data and to ensure a robust and unbiased evaluation of our model, a stratified k-fold cross-validation technique was used by splitting the remaining 80% of the dataset into subsets to train and validate the model iteratively, k times. In this technique, the dataset is divided into k folds while maintaining the same class distribution in each fold as the original dataset. We chose k=5 for a 5-fold cross-validation. Each fold maintained the proportion of class labels similar to that in the overall dataset. This helped prevent the model from being biased toward the majority class [29].

We conducted a series of experiments to determine the optimal hyperparameters for our model. These included exploring various values for learning rates, weight decay, batch sizes, and the number of training epochs [22]. Our goal was to identify the combination of hyperparameters that produced the best model performance with the highest F1-score. The initial hyperparameters were selected based on the pretrained BioMedBERT model, and they were changed during the training process iteratively. The final model hyperparameters include a batch size of 32 and a learning rate of 0.00003098.

A regularization term is added to the loss function during training to penalize large weights and prevent overfitting [29]. The modified loss function with weight decay is calculated as follows:


$$\text{Total Loss}=\text{Original Loss}+\frac{\lambda }{2}\sum_{i}{\left\|\theta_{i}\right\|}^{2}$$

Where original loss is the loss without regularization, λ is the weight decay hyperparameter, and $\Sigma_{i}{\left\|\theta_{i}\right\|}^{2}$ represents the sum of squared weights across all layers of the model.

The value of the weight decay hyperparameter is a crucial aspect of training. It determines the strength of the regularization effect. Too small a value may not prevent overfitting, while too large a value may penalize weights too much and stop the learning process. Moreover, early stopping was applied to the classifier head to prevent overfitting and improve efficiency with a patience of 5 epochs.

By following these steps, we developed a fine-tuned BioMedBERT model capable of classifying publications into the specified categories. This model was rigorously trained, validated, and optimized to maximize the classification accuracy and F_1_-score.

## Results

To assess each model for generalizability, we evaluated its performance on the test set containing 20% of the final dataset that was randomly stratified and had not been introduced to the model previously.

### Baseline Model Performance

After generating features using a FastText model and training the logistic regression model, we evaluated its performance across all classes. While the model demonstrated good performance on the majority class, indicating its efficacy in capturing prevalent patterns within the dataset, its performance on the minority class, IRGT, and SWGRT is suboptimal. Table 1 presents the baseline model’s performance across all classes, while Table 2 depicts the confusion matrix of the baseline model. The weighted average for precision, recall or sensitivity, specificity, and F_1_-score is 0.85.

**Table 1.** Performance metrics of logistic regression model across all classes.

Class	Accuracy	Precision	Recall or sensitivity	Specificity	F_1_-score
Negative	0.85	0.87	0.93	0.90	0.90
GRT^a^		0.83	0.81	0.90	0.82
IRGT^b^		0.65	0.45	0.96	0.53
SWGRT^c^		1	0.70	0.98	0.82

a GRT: group- or cluster-randomized trial.

b IGRT: individually randomized group-treatment.

c SWGRT: stepped wedge group or cluster-randomized trial.

**Table 2.** Confusion matrix.

Actual	Predicted			
Classes	Negative	GRT	IRGT	SWGRT
Negative	508	27	12	0
GRT^a^	60	269	2	2
IRGT^b^	17	15	26	0
SWGRT^c^	2	14	0	37

a GRT: group- or cluster-randomized trial.

b IGRT: individually randomized group-treatment.

c SWGRT: stepped wedge group or cluster-randomized trial.

### Pretrained Versus Fine-Tuned Performance

The low performance of the pretrained BioMedBERT classifier on all classes justifies the fine-tuning of the transformer-based model. Following the fine-tuning process using a training set that was curated by domain experts iteratively, the model’s performance exhibited noticeable enhancement in all performance metrics. The inclusion of domain expertise in the data curation process contributed to refining the model’s understanding and, consequently, improving its predictive capabilities.

Table 3 shows the performance of the latest version of the fine-tuned model on our test set, which was not seen by the model during training and validation. The confusion matrix, depicted in Table 4, provides a breakdown of the model’s predictions against the actual values. The weighted average for precision, recall or sensitivity, specificity, and F_1_-score are 0.94.

**Table 3.** Performance metrics of fine-tuned BioMedBERT across all classes.

Class	Accuracy	Precision	Recall or sensitivity	Specificity	F_1_-score
Negative	0.94	0.96	0.95	0.93	0.95
GRT^a^		0.95	0.94	0.90	0.94
IRGT^b^		0.69	0.81	0.97	0.75
SWGRT^c^		0.96	0.96	0.99	0.96

a GRT: group- or cluster-randomized trial.

b IGRT: individually randomized group-treatment.

c SWGRT: stepped wedge group or cluster-randomized trial.

**Table 4.** Confusion matrix.

Actual	Predicted			
Classes	Negative	GRT	IRGT	SWGRT
Negative	518	12	17	0
GRT^a^	14	311	4	2
IRGT^b^	8	3	47	0
SWGRT^c^	0	2	0	51

a GRT: group- or cluster-randomized trial.

b IGRT: individually randomized group-treatment.

c SWGRT: stepped wedge group or cluster-randomized trial.

## Discussion

### Principal Results

The model developed in this research used data provided by the Office of Disease Prevention at the National Institute of Health and leveraged a transformer-based pretrained language model, BioMedBERT, to identify publications of clinical trials that used one of three nested designs. The model outperformed the baseline model developed using features generated by BioWordVec and logistic regression. Compared to our baseline model, the recall and specificity for each class demonstrated improvements as follows: 2 points for recall and 3 points for specificity in non-randomized class; 13 points for recall and 0 points for specificity in GRT; 36 points for recall and 1 point for specificity in IRGT; 26 points for recall and 1 point for specificity in SWGRT. The IRGT class exhibited the lowest sensitivity compared to other classes, due to fewer examples in the training data and the inherent difficulty in identifying such publications by only processing titles and abstracts. Even for human curators, labeling these publications is challenging, often necessitating meticulous examination of the whole paper’s content. Since the fine-tuned model was trained solely on titles and abstracts, it had limited information available for predicting IRGT publications.

### Comparison With Prior Work

While machine learning and natural language processing techniques have been utilized to identify RCTs in the medical literature [15], there have been fewer models specifically designed to identify special categories of group randomized clinical trials. Existing models for biomedical document classification predominantly rely on static embedding techniques, such as Word2Vec or FastText, although recent approaches have increasingly adopted nonstatic embedding methods, particularly transformer-based models like BERT and BioBERT, for more dynamic and context-aware text representations [30-32]. Our model leverages attention mechanisms and dynamic embedding techniques to capture the varying importance of words within the context of each document. By dynamically adjusting the embedding representations based on the context of the input sequence, our model can better capture the nuances and semantic relationships within the text, leading to improved classification performance.

### Future Work

Moving forward, future research endeavors may focus on refining the model to distinguish between subcategories within each main category, such as the method, protocol, and results [33]. Tailoring the model to address these distinctions could further enhance its utility in biomedical text classification tasks, facilitating more precise and comprehensive literature analysis.

### Conclusions

Our study presents a robust framework leveraging transformer-based language models to effectively identify distinct categories of clinical trial publications within the biomedical literature. Through fine-tuning the pretrained BioMedBERT model, we achieved high accuracy and F_1_-score metrics across three categories: GRTs, IRGTs, and SWGRTs. The developed framework outperforms conventional search queries, providing advanced language understanding capabilities for discerning a broader spectrum of publications.

Our findings underscore the significance of transformer-based models in biomedical text classification, offering improved performance compared to traditional machine learning approaches and static embedding techniques. By continually updating and refining our model with new training data, we anticipate ongoing improvements in performance and adaptability over time. This iterative approach ensures the model remains up to date on the latest developments in the biomedical field, contributing to more efficient literature exploration, information retrieval, and knowledge discovery.

## Supplementary material

10.2196/63267 Multimedia Appendix 1 Python code

## Abbreviations

GRT: group- or cluster-randomized trial
IGRT: individually randomized group-treatment
SMOTE: synthetic minority over-sampling technique
SWGRT: stepped wedge group or cluster-randomized trial
