# Supplementary material for: Transformer-Based Language Models for Group Randomized Trial Classification in Biomedical Literature: Model Development and Validation
Source: JMIR Med Inform. 2025 May 9;13:e63267. doi: 10.2196/63267 (PMC12148241; doi:10.2196/63267)
Supplement: Multimedia Appendix 1 [file medinform-v13-e63267-s001.docx]

# Fine-tuning BioMedBERT for document classification using Trainer API (Publication Classifier)

# Objective:

A framework is developed to detect GRT (Group Randomized Trial), IRGT (Individually Randomized-Group Trial), and SWGRT (Stepped-wedge Group Randomized Trial) publications within a vast corpus of biomedical publications.

# Resources:

[Hugging Face Transformers](https://github.com/huggingface/transformers) - Repository for the Hugging Face Transformers library

[BiomedBERT, a pre-trained language model fine-tuned on biomedical text](https://huggingface.co/microsoft/BiomedNLP-BiomedBERT-base-uncased-abstract)

[BioMedBERT paper](https://arxiv.org/pdf/2007.15779.pdf)

[Microsoft article on training process](https://www.microsoft.com/en-us/research/blog/domain-specific-language-model-pretraining-for-biomedical-natural-language-processing/)

[PyTorch](https://pytorch.org/docs/stable/torch.html)

# BioMedBERT Overview

BioMedBERT is a pre-trained language model trained for biomedical text processing, introduced in [this paper](https://arxiv.org/pdf/2007.15779.pdf). It is a specialized variant of the BERT (Bidirectional Encoder Representations from Transformers) architecture, designed to capture the domain-specific nuances in biomedical literature.

The architecture of BioMedBERT enables it to learn contextualized representations of words and phrases bidirectionally, and understanding the contextual relationships within biomedical texts.

BioMedBERT was trained by researchers from Microsoft Research. They trained the model using abstracts from PubMed and full-text articles from PubMedCentral. This training process aimed to create a language model specifically tailored to biomedical text processing tasks.

The pre-trained BioMedBERT uses the bidirectional transformer architecture with several layers of self-attention mechanisms. The model embeddings, including word embeddings and positional embeddings contribute to encode patterns in biomedical language. During the pre-training phase, BioMedBERT is initialized with weights obtained from training on domain-specific corpus.

The model is trained on 14 million abstracts, 3.2 billion words.

This large-scale training ensures that the model captures a wide range of biomedical concepts, terminology, and contextual relationships. BERT-BASE with 12 transformer layers and 100 million parameters is used to pre-train BioMedBERT; however experiments suggest that BERT-LARGE may yield improved performance.

A pooling layer is introduced atop the transformer final layer, known as the embedding layer. This embedding layer undergoes pooling to derive a fixed-size representation of the entire input sequence. To capture non-linearity and intricate patterns within the data, a feedforward layer is incorporated. This layer is linked to the output layer, responsible for computing logits.

Importing libraries

import numpy as np

import pandas as pd

import os

from sklearn.model_selection import train_test_split, StratifiedKFold

from sklearn.metrics import (accuracy_score, precision_score, recall_score, f1_score,

classification_report, confusion_matrix)

import transformers

from transformers import (AutoTokenizer , AutoModelForSequenceClassification, TrainingArguments,

Trainer, pipeline, DataCollatorWithPadding, AutoModelForSeq2SeqLM,

EarlyStoppingCallback, IntervalStrategy, pipeline)

from datasets import load_dataset, Dataset

from evaluate import load

import torch

from GPUtil import showUtilization as gpu_usage

from numba import cuda

from torch.utils.tensorboard import SummaryWriter

import random

import wandb

import collections

import mlflow

from mlflow.tracking import MlflowClient

import torch.nn as nn

Checking the GPU information

!nvidia-smi

Setting the cuda as default

#set device

if torch.cuda.is_available():

# Tell PyTorch to use the GPU.

device = torch.device("cuda")

print('There are %d GPU(s) available.' % torch.cuda.device_count())

print('We will use the GPU:', torch.cuda.get_device_name(0))

else:

print('No GPU available, using the CPU instead.')

device = torch.device("cpu")

Importing training, validation and test data

#loading data

def load_data(train_file_path, valid_file_path):

if train_file_path and valid_file_path:

dataset = load_dataset("csv", data_files = {'train': train_file_path, 'validation': valid_file_path})

elif not valid_file_path:

dataset = load_dataset("csv", data_files = {'train': train_file_path})

return dataset

#training and k-fold validating with whole data + whole 2021 data + first pass 2022 data + second pass 2022

dataset = load_data(['RT_s.csv', 'RT_train_2021_s.csv', 'RT_test_2021_s.csv', 'RT_train_2022_s.csv', 'RT_test_2022_s.csv', 'RT_2022_s_complimentary.csv','RT_train_2023_s.csv'], None)

model_checkpoint = 'microsoft/BiomedNLP-PubMedBERT-base-uncased-abstract-fulltext'

tokenizer = AutoTokenizer.from_pretrained(model_checkpoint)

#initialize the model

def model_init():

model = AutoModelForSequenceClassification.from_pretrained(

model_checkpoint,

num_labels=4,

#id2label={index: label for index, label in enumerate(labels.names)},

#label2id={label: index for index, label in enumerate(labels.names)}

)

return model.to(device)

model = model_init()

# Function to customize data collator for training

def customized_data_collator(examples):

titles = [example['Title'] for example in examples]

abstracts = [example['Abstract'] for example in examples]

tokenized_title = tokenizer(titles , padding = 'max_length', truncation=True, max_length=30)

tokenized_abstract = tokenizer(abstracts, padding = 'max_length', truncation=True, max_length=256)

inputs = {

'input_ids' : [a+b for a,b in zip(tokenized_title['input_ids'],tokenized_abstract['input_ids'])],

'attention_mask': [a+b for a,b in zip(tokenized_title['attention_mask'],tokenized_abstract['attention_mask'])],

'token_type_ids' : [a+b for a,b in zip(tokenized_title['token_type_ids'],tokenized_abstract['token_type_ids'])]

}

label = [example['labels'] for example in examples]

batch = {

'input_ids': torch.tensor(inputs['input_ids']),

'attention_mask': torch.tensor(inputs['attention_mask']),

'labels': torch.tensor(label)

}

return batch

# Function to compute metrics

def compute_metrics_fn(eval_preds):

res = dict()

accuracy_metric = load('accuracy')

precision_metric = load('precision')

recall_metric = load('recall')

f1_metric = load('f1')

logits = eval_preds.predictions

labels = eval_preds.label_ids

preds = np.argmax(logits, axis=-1)

report = classification_report(labels, preds)

cm = confusion_matrix(labels, preds)

print(report)

print('confusion matrix: ', cm)

res.update(accuracy_metric.compute(predictions=preds, references=labels))

res.update(precision_metric.compute(predictions=preds, references=labels, average='macro'))

res.update(recall_metric.compute(predictions=preds, references=labels, average='macro'))

res.update(f1_metric.compute(predictions=preds, references=labels, average='macro'))

return res

A customized loss function is implemented to address class imbalance issue within the training data

#train final model with all data - test with early stopping , return to normal if did not work, add class weights

class CustomTrainer(Trainer):

def calculate_class_weights(training_set):

labels = [set(training_set)]

class_distribution = [0]*len(labels)

for i in labels:

class_distribution[i] = training_set.count(i)

weights = []

class_distribution = np.array(class_distribution)

num_classes = len(labels)

weight = np.sum(class_distribution)/(num_classes * class_distribution)

return weight

def compute_custom_loss(model, inputs, return_outputs=False):

target = inputs.get('labels')

weights = calculate_class_weights(target)

ce_loss = nn.CrossEntropyLoss(weight=torch.tensor(weights))

outputs = model(*inputs)

logits = outputs.get('logits')

probs = nn.functional.softmax(logits, dim = -1)

loss = ce_loss(probs.view(-1, model.config.num_labels), target.view(-1))

return (loss, outputs) if return_outputs else loss

def train(train_set, validation_set, learning_rate, weight_decay, run_name, epochs, batch_size, mode):

mlflow.start_run()

# Log training parameters

mlflow.log_param("learning_rate", learning_rate)

mlflow.log_param("batch_size", batch_size)

mlflow.log_param("num_epochs", epochs)

common_training_args = {

'output_dir': run_name,

'num_train_epochs': epochs,

'learning_rate': learning_rate,

'weight_decay': weight_decay,

'per_device_train_batch_size': batch_size,

'remove_unused_columns': False,

'run_name': run_name,

'fp16': True,

'seed': 42,

}

mode_specific_args = {}

if mode == 'train&evaluate':

mode_specific_args.update({

'logging_strategy': 'epoch',

'evaluation_strategy': 'epoch',

'save_strategy' : 'epoch',

'load_best_model_at_end' : True,

#'eval_steps': 1,

'metric_for_best_model': 'eval_f1',

})

elif mode == 'train&save':

mode_specific_args.update({

'logging_strategy': 'no',

'evaluation_strategy': 'no',

'save_strategy' : 'epoch',

'save_total_limit': 1

})

training_args = {**common_training_args, **mode_specific_args}

trainer = CustomTrainer(

model_init=model_init,

args=TrainingArguments(**training_args),

train_dataset=train_set,

data_collator=customized_data_collator,

compute_metrics=compute_metrics_fn,

eval_dataset=validation_set if mode == 'train&evaluate' else None,

#callbacks=[EarlyStoppingCallback(early_stopping_patience=5)] if mode == 'train&evaluate' else None

)

trainer.train()

if mode == 'train&evaluate':

eval_result = trainer.evaluate()

elif mode == 'train&save':

trainer.save_model(run_name)

eval_result = None

mlflow.end_run()

return eval_result

A k-fold stratified cross validation method is used to split data to training, validation and test set, ensuring that the distribution of each fold closely resembles that of the original dataset.

#5-fold stratified sampling to make sure enough sample from each class exists in k_fold and test set

def split_data(dataset):

mlflow.end_run()

# Set the experiment path

experiment_path = "PubClassifier"

# Set the experiment

mlflow.set_experiment(experiment_path)

##stratified k_fold cross validation for imbalanced dataset

skf = StratifiedKFold(n_splits=5, shuffle=True, random_state=42)

#split data, 20% for heldout_set - remaining to train and validate data

k_fold , test = next(skf.split(dataset['train']['Title'], dataset['train']['labels']))

test_set = []

for i in test:

data = {

'PMID': dataset['train']['PMID'][i],

'Title': dataset['train']['Title'][i],

'Abstract': dataset['train']['Abstract'][i],

'labels': dataset['train']['labels'][i]

}

test_set.append(data)

train_valid_set = []

for i in k_fold:

data = {

'PMID': dataset['train']['PMID'][i],

'Title': dataset['train']['Title'][i],

'Abstract': dataset['train']['Abstract'][i],

'labels': dataset['train']['labels'][i]

}

train_valid_set.append(data)

return train_valid_set, test_set

train_valid_set, test_set = split_data(dataset)

#This block runs experiments to find best hyperparameters using k fold corss validation

def tuning_lm(train_valid_set, learning_rate, weight_decay, run_name, epochs, batch_size):

skf = StratifiedKFold(n_splits=5, shuffle=True, random_state=42)

for i, (train_idx , valid_idx) in enumerate(skf.split([entry['Title'] for entry in train_valid_set], [entry['labels'] for entry in train_valid_set])):

train_set = []

valid_set = []

eval_result = []

for j in train_idx:

data = {

'PMID': train_valid_set[j]['PMID'],

'Title': train_valid_set[j]['Title'],

'Abstract': train_valid_set[j]['Abstract'],

'labels': train_valid_set[j]['labels']

}

train_set.append(data)

for k in valid_idx:

data = {

'PMID': train_valid_set[k]['PMID'],

'Title': train_valid_set[k]['Title'],

'Abstract': train_valid_set[k]['Abstract'],

'labels': train_valid_set[k]['labels']

}

valid_set.append(data)

train_set = [{key: tensor.to(device) if isinstance(tensor, torch.Tensor) else tensor for key, tensor in sample.items()} for sample in train_set]

valid_set = [{key: tensor.to(device) if isinstance(tensor, torch.Tensor) else tensor for key, tensor in sample.items()} for sample in valid_set]

print(f"The model performance on fold {i+1}:")

train(train_set, valid_set, learning_rate, weight_decay, run_name, epochs, batch_size, 'train&evaluate')

#final hyperparameters

# wd = 0

# lr = 3.098e-5

# epoch = 4 or 5

# batch = 32

tuning_lm(train_valid_set,3.098e-5, 0, 'pubmedbert', 5, 32)

tuning_lm(train_valid_set,3.098e-5, 0, 'pubmedbert_1', 10, 32)

tuning_lm(train_valid_set,3.098e-5, 0, 'pubmedbert_2', 5, 16)

tuning_lm(train_valid_set,3.098e-5, 0, 'pubmedbert_3', 10, 16)

def eval_performance(train_valid_set, test_set, learning_rate, weight_decay, run_name, epochs, batch_size):

train_valid_set = [{key: tensor.to(device) if isinstance(tensor, torch.Tensor) else tensor for key, tensor in sample.items()} for sample in train_valid_set]

test_set = [{key: tensor.to(device) if isinstance(tensor, torch.Tensor) else tensor for key, tensor in sample.items()} for sample in test_set]

train(train_valid_set, test_set, learning_rate, weight_decay, "pubmedbert_test", epochs, batch_size, 'train&evaluate')

eval_performance(train_valid_set, test_set, 3.098e-5, 0, 'pubmed_5_16', 5, 16)

eval_performance(train_valid_set, test_set, 3.098e-5, 0, 'pubmed_10_16', 10, 16)

def save_candidate(all_data_path, learning_rate, weight_decay, run_name, epochs, batch_size):

dataset = load_dataset("csv", data_files = {'unknown': all_data_path})

all_data = []

for i, _ in enumerate(dataset['unknown']['PMID']):

data = {

'Title': dataset['unknown']['Title'][i] if not pd.isna(dataset['unknown']['Title'][i]) else 'No text available',

'Abstract': dataset['unknown']['Abstract'][i] if not pd.isna(dataset['unknown']['Abstract'][i]) else 'No text available',

'labels': dataset['unknown']['labels'][i]

}

all_data.append(data)

all_data = [{key: tensor.to(device) if isinstance(tensor, torch.Tensor) else tensor for key, tensor in sample.items()} for sample in all_data]

print(len(all_data))

train(all_data, None, learning_rate, weight_decay, run_name, epochs, batch_size, 'train&save')

save_candidate(['RT_s.csv', 'RT_train_2021_s.csv', 'RT_test_2021_s.csv', 'RT_train_2022_s.csv', 'RT_test_2022_s.csv', 'RT_2022_s_complimentary.csv'], 3.098e-5, 0, 'candidate_PubMedBERT2022_02-02-24', 5, 32)

This block is used to identify grt, irgt, swgrt publications from corpus

#inference - generate predictions for unknown data using fine-tuned model

def inference(candidate_model_path, unknown_file_path, output_predictions):

model_checkpoint = 'microsoft/BiomedNLP-PubMedBERT-base-uncased-abstract-fulltext'

ft_model = AutoModelForSequenceClassification.from_pretrained(candidate_model_path).to(device)

tokenizer = AutoTokenizer.from_pretrained(model_checkpoint)

dataset = load_dataset("csv", data_files={'unknown': unknown_file_path})

#dataset['unknown'] = dataset['unknown'][:100]

results = collections.defaultdict(list)

batch_size = 100

for start in range(0, len(dataset['unknown']['PMID']), batch_size):

end = min(start + batch_size, len(dataset['unknown']['PMID']))

batch_data = []

for i in range(start, end):

data = {

'PMID': dataset['unknown']['PMID'][i],

'Title': dataset['unknown']['Title'][i] if not pd.isna(dataset['unknown']['Title'][i]) else 'No text available',

'Abstract': dataset['unknown']['Abstract'][i] if not pd.isna(dataset['unknown']['Abstract'][i]) else 'No text available',

}

batch_data.append(data)

titles = [example['Title'] for example in batch_data]

abstracts = [example['Abstract'] for example in batch_data]

pmids = [example['PMID'] for example in batch_data]

tokenized_title = tokenizer(titles, padding='max_length', truncation=True, max_length=50)

tokenized_abstract = tokenizer(abstracts, padding='max_length', truncation=True, max_length=256)

inputs = {

'input_ids': [a + b for a, b in zip(tokenized_title['input_ids'], tokenized_abstract['input_ids'])],

'attention_mask': [a + b for a, b in zip(tokenized_title['attention_mask'], tokenized_abstract['attention_mask'])],

'token_type_ids': [a + b for a, b in zip(tokenized_title['token_type_ids'], tokenized_abstract['token_type_ids'])]

}

# Create input tensors

input_ids = torch.tensor(inputs['input_ids']).to(device)

attention_mask = torch.tensor(inputs['attention_mask']).to(device)

token_type_ids = torch.tensor(inputs['token_type_ids']).to(device)

with torch.no_grad():

outputs = ft_model(input_ids=input_ids, attention_mask=attention_mask, token_type_ids=token_type_ids)

predictions = outputs.logits.argmax(dim=1).tolist()

# Update results dictionary with predictions

for pm, title, abstract, pred in zip(pmids, titles, abstracts, predictions):

results[pm].append(pred)

results[pm].append(title)

results[pm].append(abstract)

# Convert results to DataFrame and save to CSV

df = pd.DataFrame.from_dict(results, orient='index')

df.to_csv(output_predictions, index=True)

return results

predictions = inference('candidate_PubMedBERT2022_06-02-24-2/checkpoint-255', '2023unknownpubs.csv', '2023publications_test.csv')

ft_model = AutoModelForSequenceClassification.from_pretrained("candidate_PubMedBERT2022_06-02-24-2/checkpoint-255")

print(ft_model)
